# MgO Nanoparticles-Incorporated PCL/Gelatin-Derived Coaxial Electrospinning Nanocellulose Membranes for Periodontal Tissue Regeneration

**DOI:** 10.3389/fbioe.2021.668428

**Published:** 2021-03-25

**Authors:** Wenzao Peng, Shuangshuang Ren, Yibo Zhang, Ruyi Fan, Yi Zhou, Lu Li, Xuanwen Xu, Yan Xu

**Affiliations:** ^1^Jiangsu Key Laboratory of Oral Diseases, Nanjing Medical University, Nanjing, China; ^2^Department of Periodontics, Affiliated Hospital of Stomatology, Nanjing Medical University, Nanjing, China; ^3^Jiangsu Province Engineering Research Center of Stomatological Translational Medicine, Nanjing, China; ^4^State Key Laboratory of Pharmaceutical Biotechnology, Department of Sports Medicine and Adult Reconstructive Surgery, Nanjing Drum Tower Hospital, The Affiliated Hospital of Nanjing University Medical School, Nanjing, China

**Keywords:** nanocellulose membranes, electrospinning, polycaprolactone, MgO nanoparticle, periodonditis

## Abstract

Electrospinning technique has attracted considerable attention in fabrication of cellulose nanofibrils or nanocellulose membranes, in which polycaprolactone (PCL) could be used as a promising precursor to prepare various cellulose nanofibril membranes for periodontal tissue regeneration. Conventional bio-membranes and cellulose films used in guided tissue regeneration (GTR) can prevent the downgrowth of epithelial cells, fibroblasts, and connective tissue in the area of tooth root but have limitations related to osteogenic and antimicrobial properties. Cellulose nanofibrils can be used as an ideal drug delivery material to encapsulate and carry some drugs. In this study, magnesium oxide (MgO) nanoparticles-incorporated PCL/gelatin core-shell nanocellulose periodontal membranes were fabricated using coaxial electrospinning technique, which was termed as Coaxial-MgO. The membranes using single-nozzle electrospinning technique, namely Blending-MgO and Blending-Blank, were used as control. The morphology and physicochemical property of these nanocellulose membranes were characterized by scanning electron microscopy (SEM), energy-dispersive spectrum of X-ray (EDS), transmission electron microscopy (TEM), contact angle, and thermogravimetric analysis (TGA). The results showed that the incorporation of MgO nanoparticles barely affected the morphology and mechanical property of nanocellulose membranes. Coaxial-MgO with core-shell fiber structure had better hydrophilic property and sustainable release of magnesium ion (Mg^2+^). CCK-8 cell proliferation and EdU staining demonstrated that Coaxial-MgO membranes showed better human periodontal ligament stem cells (hPDLSCs) proliferation rates compared with the other group due to its gelatin shell with great biocompatibility and hydrophilicity. SEM and immunofluorescence assay results illustrated that the Coaxial-MgO scaffold significantly enhanced hPDLSCs adhesion. *In vitro* osteogenic and antibacterial properties showed that Coaxial-MgO membrane enhanced alkaline phosphatase (ALP) activity, formation of mineralized nodules, osteogenic-related genes [ALP, collagen type 1 (COL1), runt-related transcription factor 2 (Runx2)], and high antibacterial properties toward *Escherichia coli* (*E. coli*) and *Actinobacillus actinomycetemcomitans* (*A. a*) when compared with controls. Our findings suggested that MgO nanoparticles-incorporated coaxial electrospinning PCL-derived nanocellulose periodontal membranes might have great prospects for periodontal tissue regeneration.

## Introduction

Periodontitis is one of the most ubiquitous chronic oral diseases caused by trauma or various kinds of oral pathogens infection. The major clinical manifestations of periodontitis include the formation of periodontal pockets and alveolar bone resorption, which eventually lead to progressive loss of alveolar bone and teeth (Dentino et al., 2013; Larsson et al., 2016; Liu J. et al., 2019). Consequently, multifunctional membranes with osteogenic and antibacterial properties for periodontal tissue regeneration are highly desirable but also a great challenge to manufacture (Graziani et al., 2017). Among various therapeutic options, the GTR technique (Oortgiesen et al., 2012; Needleman et al., 2019; Stavropoulos et al., 2020), in which membranes are employed as a barrier to prevent the downgrowth of epithelial cells, fibroblasts, and connective tissue in the area of the tooth root, has been widely used in periodontal diseases (Zwahlen et al., 2009; Dentino et al., 2013; Larsson et al., 2016; Liang et al., 2020). The most commonly used membranes in GTR, like Bio-Gide^®^, have excellent biocompatibility but have limitations in terms of osteogenic and antimicrobial properties. Infection caused by numbers of bacteria may result in unsuccessful treatment. Therefore, considerable attention has been focused on the development of multifunctional membranes for periodontal tissue regeneration (Li et al., 2016; Liang et al., 2020).

To date, a great number of preparation methods have been developed to constitute GTR membranes, among which the electrospinning technique has attracted considerable attention due to its advantage in fabricating nanocellulose membranes simply and versatilely (Szentivanyi et al., 2011; Doostmohammadi et al., 2020). The electrospun membranes possess high specific surface area and porosity, which can mimic the structure and function of the native extracellular matrix (ECM) (Szentivanyi et al., 2011; Kennedy et al., 2017; Meireles et al., 2018). Besides, the diameter and direction of electrospun nanocellulose can also be regulated to enhance attachment, proliferation, migration, and differentiation of biological cells (Lim and Mao, 2009; Lee et al., 2019; Parham et al., 2020). One advantage of electrospinning is its versatility in directly incorporating bioactive agents, including anti-cancer drugs, nanoparticles, growth factors, into the nanocellulose matrix without complicated processes (Xue et al., 2014; Ren et al., 2017). However, a specific disadvantage that should be considered while dealing with the drug delivery system is its high initial release that may cause irreversible damage to the surrounding cells or tissues (Khalf and Madihally, 2017; De-Paula et al., 2019; Pant et al., 2019). To address this issue, the coaxial electrospun fiber composed of the shell layer and the core layer was introduced to fabricate the bioactive agents-incorporated cellulose membranes (Dayem et al., 2016; Si et al., 2016; Hickey et al., 2017; Ke et al., 2019). The core-shell nanocellulose matrix exhibits a specific advantage whereby the bioactive agents can be restricted to the core layer, alleviating their initial burst release (Lin et al., 2018). In addition, the release rate of bioactive agents can also be controlled by tuning the composition and thickness of the shell, thus avoiding irreversible damage or undesired side effects to surrounding tissues (Li et al., 2013; Khalf and Madihally, 2017). Among the various materials used for coaxial electrospinning, PCL is considered to be a very promising precursor for electrospun cellulose nanofiber due to its good biocompatibility, mechanical properties, and ease of fabrication into fibers. Yet, the synthetic PCL also has some drawbacks, such as limited cell affinity and poor hydrophilicity (Hiep and Lee, 2010; Qian et al., 2019). Gelatin with cell-binding sites and biomolecular signatures is the most widely used natural polymer in tissue engineering, including simulating ECM and promoting cell adhesion due to its low antigenicity, desirable biocompatibility, and biodegradability (Sridhar et al., 2014; Pant et al., 2019). Therefore, hybrid nanofibers with core-shell structure combining PCL and gelatin can be obtained by using coaxial electrospinning and subsequently used as a nanocellulose scaffold in tissue engineering (Ren et al., 2017).

Recently, nanomaterials, such as nanocellulose, nano-gel, and nano-membranes, have brought innovation in biomedical application due to their excellent biocompatibility, physicochemical properties and ease of functionalization (Nie et al., 2018a,b; Lu et al., 2020; Wang P. et al., 2020; Zhang et al., 2021). Generally, various extracts and inorganic substances that have ability to scavenge free radicals can inhibit inflammation casing by bacterias (Huang et al., 2018; Dong et al., 2020; Pei et al., 2020; Gu et al., 2021; Zheng et al., 2021), among which MgO nanoparticles have attracted considerable attention in tissue engineering due to their multifold effects on accelerating osteogenic differentiation of biological cells and inhibiting bacterial activity (Khandaker et al., 2013; Ke et al., 2019). Liu et al. (2020) reported that PLA/gelatin periodontal membrane fabricated by electrospinning biodegradable polymers with MgO nanoparticles demonstrated a dose-dependent magnesium ion-induced osteogenic activity of rabbit bone marrow stem cells (rBMSCs). Besides, numerous studies have also demonstrated that MgO nanoparticles have attracted considerable attention in biomedical applications due to their great antibacterial activities toward various kinds of bacteria (Karthik et al., 2019). Abbas’s work demonstrated that MgO nanoparticle solutions had good antimicrobial activity both *in vitro* and *in vivo* with minimal toxicity (Monzavi et al., 2014). Hence, MgO-incorporated coaxial electrospun nanocellulose membrane could be very promising platforms for periodontal tissue regeneration.

In this work, MgO nanoparticles-incorporated PCL/gelatin nanocellulose membranes were fabricated using coaxial electrospinning method, and their morphological and physicochemical features were characterized by TEM, SEM, contact angle, and TGA. Then, comprehensive assessments were carried out to investigate their effect on proliferation, attachment, and osteogenic differentiation of hPDLSCs. Antibacterial properties of as-prepared nanocellulose membranes were evaluated by prohibiting the growth of *Escherichia coli* (*E. coli*) and *Actinobacillus actinomycetemcomitans* (*A. a*). Thus, we speculated that the MgO nanoparticles-incorporated PCL/gelatin nanocellulose membranes fabricated by coaxial electrospinning with excellent osteogenic and antibacterial properties could provide valuable insights into the development of bio-membranes for periodontal regeneration.

## Materials and Methods

### Materials

Polycaprolactone (Mw = 160000) was purchased from Jinan Daigang Biomaterial Co., Ltd, Jinan, China. Gelatin powder (type A) (Mw = 80000) was obtained from Sigma-Aldrich Co., Ltd, China. MgO nanoparticles were purchased from Shanghai Bike New Material Technology Co., Ltd, China. 1,1,1,3,3,3-Hexafluoro-2-propanol (HFIP) was purchased from Aladdin Co., Ltd, China. Cell-Light^TM^ EdU DNA Cell Proliferation Kit was obtained from RiboBio Co., Ltd, China. Cell Counting Kit-8 (CCK-8) was obtained from Dojindo Co., Ltd, Japan.

### Characterization

The morphology of MgO nanoparticles was characterized by transmission electron microscopy (JEOL 1200EX). Surface morphology, topography and energy dispersive spectrometer (EDS) analysis of PCL/gelatin nanocellulose membranes were characterized by field-emission SEM (FEI Nova NanoSEM450). The contact angle of PCL/gelatin nanocellulose membranes was carried out by static water contact angle measurement (Automatic Contact Angle Meter Model SL200B, China). The mechanical property of PCL/gelatin nanocellulose membranes was measured by an electronic universal testing machine (WSM).

### Fabrication of PCL/Gelatin Nanocellulose Membranes

Polycaprolactone/gelatin nanocellulose membranes were prepared by the electrospinning method. As for coaxial electrospinning, PCL and gelatin were dissolved in HFIP according to the proportion in Table 1 to form core solution and shell solution, respectively. The outer and inner tube had a diameter of 1.11 and 0.34 mm, respectively. A constant-volume flow rate of 0.5 ml/h for core solution and 2.5 ml/h for shell solution were accomplished via two syringe pumps. An electrostatic field force was created by applying a current of 20–22 kV voltages, which introduced the formation of nanofibers from the solution. In addition, the distance between the nozzle tip and collector plate was set up to 18 cm. MgO nanoparticles 3% (w/v) were added to the core solution under magnetic stirring at room temperature for 72 h. As a control, the traditional single fiber membrane loaded with the same amount of MgO nanoparticles was also performed (the parameters were shown in Table 1).

**TABLE 1:** Parameters for electrospun fibers.

	**Solvent**	**Core**		**Shell**		**Voltage (kV)**	**Time (h)**
		**Solute (w/v)**	**Feed speed (ml/h)**	**Solute (w/v)**	**Feed speed (ml/h)**		
Coaxial-Blank	HFIP	10% PCL	0.5	10% gelatin	2.5	20–22	8
Coaxial-MgO	HFIP	10% PCL + 3% MgO	0.5	10% gelatin	2.5	20–22	8
		**Solute (w/v)**		**Feed speed (ml/h)**			
Blending-Blank	HFIP	1.7% PCL + 8.3% gelatin		3		17–19	8
Blending-MgO	HFIP	1.7% PCL + 8.3% gelatin + 0.5% MgO		3		17–19	8

### Mg^2+^ Release Study

Magnesium oxide nanoparticles-incorporated PCL/gelatin nanocellulose membranes were punched into uniform disks with a diameter of 6 mm, which were incubated at 37°C with 5% CO_2_ in 48-well plates supplemented with 250 μl PBS buffer (No Calcium, No Magnesium, pH = 7.4). Then, 20 μl PBS buffer was added every 3 days to maintain the total volume, and subsequently, the amount of Mg^2+^ at a predetermined time was quantificationally measured by a Magnesium Assay Kit (Jiancheng, Nanjing).

### Cell Experiments

hPDLSCs were individually isolated from healthy premolars extracted for orthodontic treatment from teenagers aged 11–16 years without periodontitis, caries, and cracks and subsequently purified using a limited dilution method (Seo et al., 2004). The cells were identified by detecting the surface markers such as CD73, CD90, CD45, and CD34 (BD, United States) by flow cytometry (BD FACSCalibur, United States). Osteogenic differentiation and adipogenic differentiation were respectively introduced to prove the differentiation potential of the cells (Lin et al., 2008; Ng et al., 2016). Cells were cultured at 37°C with 5% CO_2_ in the growth medium, α-MEM medium (Gibco, United States) supplemented with 10% fetal bovine serum (Gibco, United States) and 1% penicillin-streptomycin solution (Gibco, United States). The medium was replaced every 3 days.

#### Cell Proliferation Assay

These sterilized PCL/gelatin nanocellulose membranes with a diameter of 6 mm were cultured with hPDLSCs at a density of 2 × 10^4^ cells at 37°C with 5% CO_2_ in 48-well plates. After incubation for 1 day, 3 days, 5 days, and 7 days, the cells were washed with PBS buffer, followed by the addition of 100 μl α-MEM medium supplemented with 10% Cell Counting Kit-8 and incubated at 37°C for 2 h. The optical density (OD) values at 450 nm was measured by a microplate reader (Spectramax190, MD, United States).

#### EdU Staining Assay

Electrospun membranes 6 mm in diameter were incubated with hPDLSCs at a density of 2 × 10^4^ cells at 37°C with 5% CO_2_ in 48-well plates for 24 h. After that, the cells were washed with PBS three times and fixed with 4% paraformaldehyde for 30 min, stained by Cell-Light^TM^ EdU DNA Cell Proliferation Kit (RiboBio, Guangzhou, China) according to the manufacturer’s instructions, and observed under a fluorescence microscope (DMI6000B, Leica, Germany).

#### Cell Attachment Assay

The sterilized PCL/gelatin nanocellulose membranes were co-cultured with hPDLSCs at a density of 4 × 10^5^ cells at 37°C with 5% CO_2_ in 6-well plates for 24 h, followed by washing with PBS buffer for three times to remove the unattached cells and fixing with 4% paraformaldehyde overnight. The cells were stained with rhodamine phalloidin for cytoskeleton and 4’, 6-Diamidino-2-phenylindole, dihydrochloride (DAPI) for the nucleus, followed by observation under inverted fluorescence microscope (DMI6000B, Leica, Germany).

#### ALP Activity and Staining

These four sterilized PCL/gelatin nanocellulose membranes with a diameter of 6 mm were incubated with hPDLSCs at 37°C with 5% CO_2_ in 48- and 24-well plates overnight respectively, after which the culture medium was replaced by osteogenic differentiation medium containing growth medium supplemented with 0.1 μM dexamethasone, 300 μM ascorbic acid and 10 mM β-glycerophosphate (Sigma, United States). After incubation for 7 days, the cells were harvested, and the ALP activity was measured by Alkaline Phosphatase Assay Kit (Beyotime Biotechnology, China) and stained by BCIP/NBT Alkaline Phosphatase Color Development Kit (Beyotime Biotechnology, China) according to the manufacturer’s instructions. The OD values at 520 nm were measured, and the wells were examined under optical microscopy (SMZ1000, Nikon, Japan) and photographed with digital camera (EOS 6D, Japan).

#### Alizarin Red Staining

These four kinds of sterilized PCL/gelatin nanocellulose membranes with a diameter of 15 mm were incubated with hPDLSCs at a density of 2 × 10^5^ cells at 37°C with 5% CO_2_ in 24-well plates overnight. After that, the cells were cultured in osteogenic differentiation medium for 14 days, stained by 2% alizarin red S (ARS, Leagene, China) staining solution for 5 min. After being washed with PBS buffer three times, the plates were observed by optical microscope (SMZ1000, Nikon, Japan). After that, mineralized nodules stained by ARS were desorbed with 10% (w/v) cetylpyridinium chloride (Sigma-Aldrich, China). We measured the absorbance at 562 nm for quantification.

#### Reverse Transcription-Quantitative Polymerase Chain Reaction (RT-qPCR)

The osteogenic-related genes, including ALP, COL1, and Runx2, were further investigated by RT-qPCR. The hPDLSCs were cultured with different kinds of sterilized PCL/gelatin nanocellulose membranes for 3 days, followed by the isolation of total RNA by the RNAsimple Total Kit (TianGen, China). After that, the cDNA was generated and analyzed by PrimeScript^TM^ RT Master Mix (Perfect Real Time) and TB Green Premix EX Taq II respectively (TaKaRa, Japan), followed by the amplification and detection by LightCycler^®^ 96 (Roche, Mannheim, Germany). The primers are listed in Table 2.

**TABLE 2:** Primer sequences.

**Primer name**	**Forward primer sequence (5′–3′)**	**Reverse primer sequence (5′–3′)**
ALP	GAGATGTTGTCCTGACACTTGTG	AGGCTTCCTCCTTGTTGGGT
RUNX2	TGGTTACTGTCATGGCGGGTA	TCTCAGATCGTTGAACCTTGCTA
COL1	GAGGGCCAAGACGAAGACATC	CAGATCACGTCATCGCACAAC
Human actin	GTCCCTCACCCTCCCAAAAG	GCTGCCTCAACACCTCAACCC

### Antibacterial Activity

These sterilized PCL/gelatin nanocellulose membranes with a diameter of 20 mm were incubated with *E. coli* (ATCC-25922) and *A. a* (ATCC-29523) at a density of 1 × 10^7^ CFU for 24 h. After that, the bacterial suspension followed by a series of dilution were swabbed to the surface of LB agar plate and Columbia blood agar respectively and were incubated at 37°C for 24 h. The number of the bacteria colonies were calculated to evaluate the antibacterial activity using a colonometer (Interscience Scan1200, France).

### Statistical Analysis

The differences between groups were determined by using one-way ANOVA followed by Tukey’s *post hoc* analysis. A *p*-value of < 0.05 was considered as statistical significance. Statistical analysis was performed with GraphPad Prism 8.

## Results

### Surface Topography of PCL/Gelatin Nanocellulose Membranes

**FIGURE 1:**
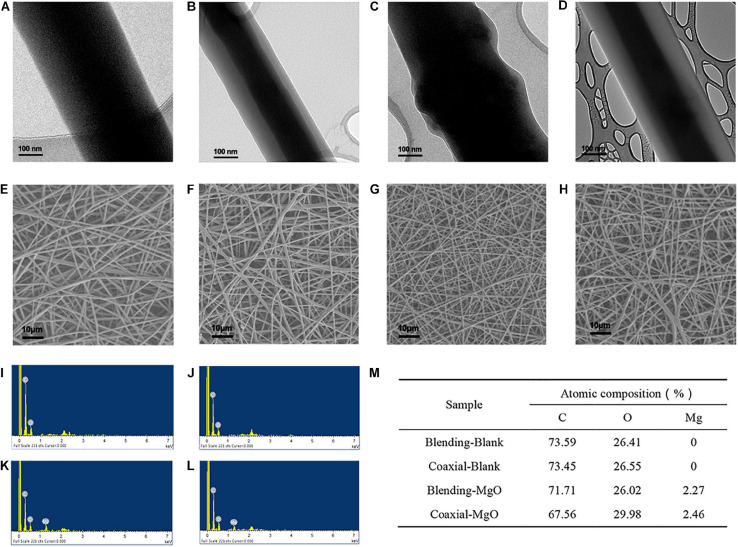
Structure and characterization of four different electrospun membranes. TEM images of **(A)** Blending-Blank, **(B)** Coaxial-Blank, **(C)** Blending-MgO, and **(D)** Coaxial-MgO. SEM images of **(E)** Blending-Blank, **(F)** Coaxial-Blank, **(G)** Blending-MgO, **(H)** Coaxial-MgO. **(I–L)** EDS spectra of Blending-Blank, Coaxial-Blank, Blending-MgO, and Coaxial-MgO, respectively. **(M)** Semi-quantitative determination of elemental composition of these electrospun fiber surfaces. C, carbon; O, oxygen; Mg, magnesium.

PCL/gelatin nanocellulose membranes were prepared by coaxial or single-nozzle electrospinning technique alone or incorporated with MgO nanoparticles, after which their morphology was characterized. As shown in Figures 1A,B, the densely compacted and core-shell structure single fiber for single-nozzle and coaxial electrospinning were observed by TEM images, respectively. The darker (core) and lighter (shell) areas represented the PCL and gelatin region, respectively. After incorporated with MgO nanoparticles, obvious lumps of MgO nanoparticles marked higher in nanocellulose membranes could be observed in both Blending-MgO and Coaxial-MgO (Figures 1C,D). The SEM micrographs for four types of PCL/gelatin nanocellulose membranes were shown in Figures 1E–H, where it could be seen that the blank nanocellulose membranes without MgO nanoparticles exhibited some web-like fine fibers with a smooth surface and high interconnected porosity. As expected, the nanocellulose membranes were evenly arranged, and no bead-like structures were observed. EDS (Figures 1I–M) was further employed to investigate the elemental composition of our electrospun membranes. In comparison with Blending-Blank and Coaxial-Blank that only contained C and O elements, the magnesium element ratio for Blending-MgO and Coaxial-MgO were 2.27 and 2.46, respectively, indicating the successful incorporation of MgO nanoparticles.

### Physicochemical Properties of PCL/Gelatin Nanocellulose Membranes

**FIGURE 2:**
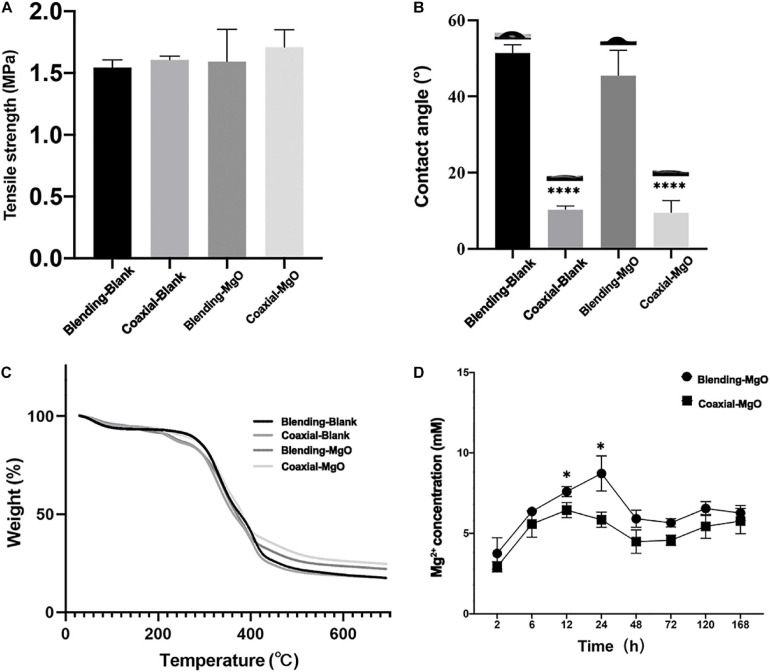
Chemical, physical, and mechanical properties of the electrospun membranes. **(A)** Tensile strength of Blending-Blank, Coaxial-Blank, Blending-MgO, and Coaxial-MgO, respectively. **(B)** Contact angles of the electrospun membranes. **(C)** Thermal stabilities of PCL/gelatin nanocellulose membranes tested by TGA. **(D)** Mg^2+^ release of Blending-MgO and Coaxial-MgO in PBS from 0 to 168 h.

Mechanical strength of PCL/gelatin nanocellulose membranes were firstly investigated using electronic universal testing machine. As shown in Figure 2A, the mean values of tensile strength for Blending-Blank, Coaxial-Blank, Blending-MgO, and Coaxial-MgO were 1.54, 1.61, 1.60, and 1.71 MPA, respectively, and there was no significant difference among these four groups (*p* > 0.05). These results indicated that the incorporation of MgO nanoparticles did not affect the tensile strength of the nanocellulose membranes.

It is well known that the hydrophilic property of the scaffold may greatly affect their interaction with biological cells, such as cell adhesion and proliferation. Hence, the surface wettability of the as-prepared PCL/gelatin nanocellulose membranes was evaluated by contact angle. As shown in Figure 2B, the average contact angles of coaxial nanocellulose membranes were much smaller than that of single fiber membranes. These results demonstrated that the coaxial structure that was made up of PCL encapsulated in gelatin increased their hydrophilic property, which was conducive to cell adhesion and provided a better physiological environment for cell growth. When MgO nanoparticles were added, a slight decrease from 10.29° (51.4°) to 9.48° (45.5°) was observed for coaxial (single fiber) nanocellulose membrane, indicating that the added MgO nanoparticles could improve the hydrophilic property of the blank PCL/gelatin nanocellulose membranes. Thermal stability of PCL/gelatin nanocellulose membranes was further tested by TGA; the obtained weight loss curves were shown in Figure 2C. Our results showed that the initial degradation temperature for coaxial nanocellulose membrane increased after incorporated MgO nanoparticles but decreased for Blending-MgO and Blending-Blank. This, in turn, suggested that the MgO nanoparticles could enhance the thermal stability of the coaxial nanocellulose membrane. When the temperature was increased to 500°C, the thermal degradation of all the four nanocellulose membranes was relatively stable, and the incorporation of MgO nanoparticles could significantly increase the maximum degradation temperature of nanocellulose membranes.

Furthermore, the accumulated release of Mg^2+^ from the Blending-MgO and Coaxial-MgO nanocellulose membranes in PBS over a period of 168 h was quantified by Magnesium Assay Kit. As shown in Figure 2D, a relatively burst initial release of Mg^2+^ within the first 24 h was observed. The results revealed higher quantities of Mg^2+^, almost beyond 10 mM within 24 h, in group Blending-MgO, which was higher than in group Coaxial-MgO. After that, Mg^2+^ was sustainably released for both Blending-MgO and Coaxial-MgO over 168 h, resulting in a stable Mg^2+^ concentration around 5 mM. Numerous studies have shown that 5–10 mM Mg^2+^ had a slight cytotoxic effect on cells and could promote cells’ osteogenic differentiation (Mangalampalli et al., 2018; Onder et al., 2018). These results indicated that the MgO-incorporated coaxial PCL/gelatin nanocellulose membranes with the sustainable release of Mg^2+^ might be suitable for accelerating osteogenic differentiation of cells.

### The Proliferation of hPDLSCs Toward PCL/Gelatin Nanocellulose Membranes

**FIGURE 3:**
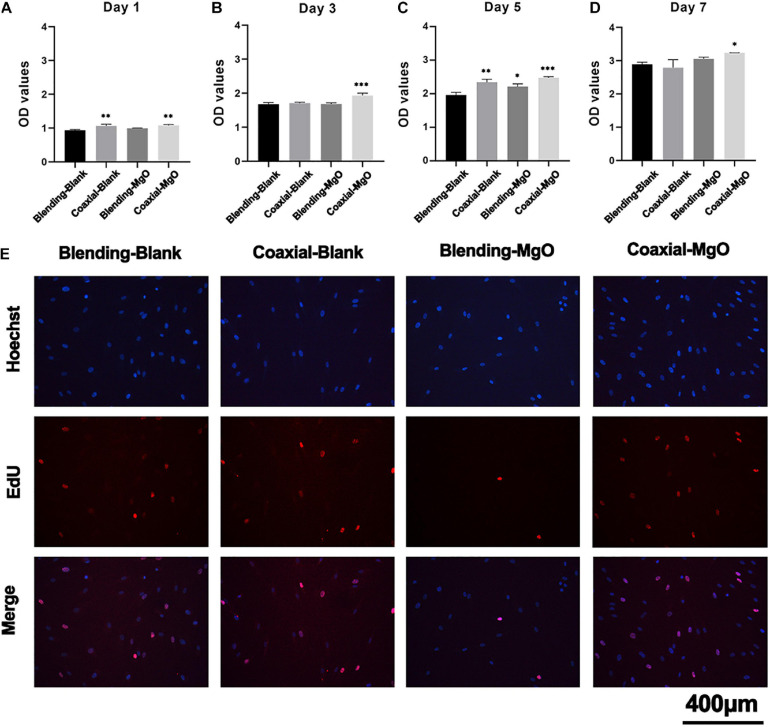
Biocompatibility of electrospun membranes. hPDLSCs were cultured on Blending-Blank, Coaxial-Blank, Blending-MgO, and Coaxial-MgO, respectively, for **(A)** 1, **(B)** 3, **(C)** 5, and **(D)** 7 days, and the cell viability was compared by the OD value using a CCK-8 kit. **(E)** Cell proliferation of hPDLSCs seeded on the electrospun membranes after 12 h were analyzed by EdU staining. Red, EdU-labeled nuclei of proliferative cells; Blue, Hoechst 33342-labeled cell nuclei.

The proliferative capability of hPDLSCs toward PCL/gelatin nanocellulose membranes was further investigated using CCK-8 and EdU assays. As illustrated in Figures 3A–D, the cell viability of hPDLSCs exceeds 100% in all groups and exhibited a time-dependent cell proliferation after co-incubation for 1, 3, 5, and 7 days. These results indicated that all the PCL/gelatin nanocellulose membranes were not cytotoxic and had excellent biocompatibility. Surprisingly, the OD values in Coaxial-MgO group were significantly higher compared to other groups, indicating that the coaxial structure with smart release of Mg^2+^ could promote the proliferation of hPDLSCs. Moreover, EdU staining (Figure 3E) also revealed that the proliferation of hPDLSCs after treatment with Coaxial-MgO was the most obvious among the four types of nanocellulose membranes because of its gelatin shell with great biocompatibility and hydrophilicity and suitable Mg^2+^ release.

### Attachment of hPDLSCs Toward PCL/Gelatin Nanocellulose Membranes

**FIGURE 4:**
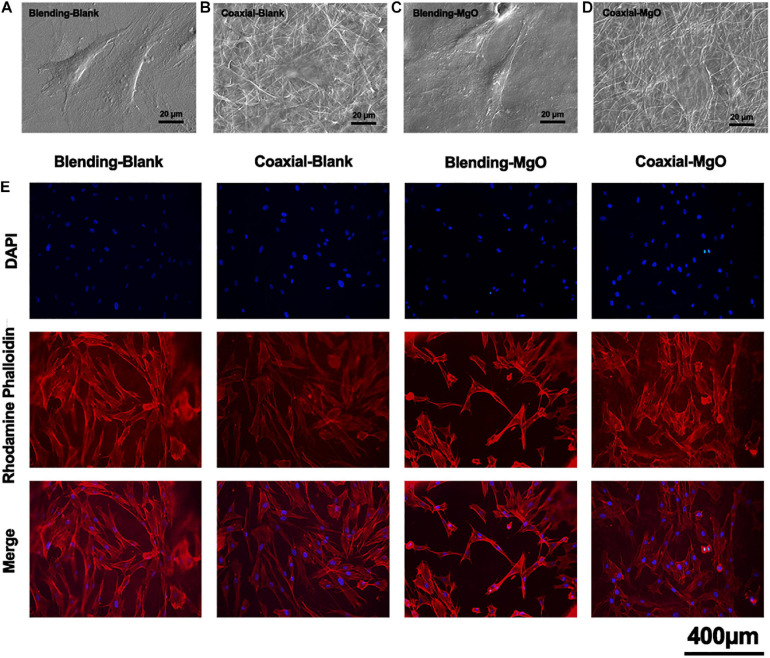
Cell morphology and attachment on the electrospun membranes. SEM micrographs of hPDLSCs cultured on **(A)** Blending-Blank, **(B)** Coaxial-Blank, **(C)** Blending-MgO, and **(D)** Coaxial-MgO for 24 h. **(E)** Fluorescent staining of hPDLSCs cultured on electrospun membranes for 24 h. Red, cytoskeleton stained by rhodamine phalloidin; Blue, nuclei stained by DAPI.

To further understand how our PCL/gelatin nanocellulose membranes affected the interaction between hPDLSCs and their environment, including cell morphology and cell attachment, SEM was employed. As shown in Figures 4A–D, after 24 h of culture, the hPDLSCs randomly spread along the fibers, and their filopodia were extended. Specifically, the surfaces of Blending-Blank (Figure 4A) and Blending-MgO (Figure 4C) membranes became smooth due to the disinfection by 75% alcohol, thus exhibiting a convex round shape of hPDLSCs. In contrast, a more flattened morphology for Coaxial-Blank (Figure 4B) and Coaxial-MgO (Figure 4D) was observed. In addition, the cytoskeletal morphology of hPDLSCs was also investigated by the inverted fluorescence microscope (Figure 4E) after 24 h in culture with our electrospinning nanocellulose membranes. The immunofluorescence images demonstrated that the morphology of hPDLSCs was narrow and filamentous, and the cytoskeleton was poorly developed in the Blending-MgO group, while strong F-actin staining and elongated filopodia were observed in Coaxial-MgO. These observations revealed the same tendency with SEM images, which was attributed to the fact that the explosive release of Mg^2+^ from Blending-MgO in a short time might decrease the attachment of hPDLSCs. In addition, the gelatin coating might also enhance the interaction between hPDLSCs and nanocellulose membranes due to the integrin-binding site of the gelatin molecule.

### Effects of PCL/Gelatin Nanocellulose Membranes on Osteogenic Differentiation of hPDLSCs

**FIGURE 5:**
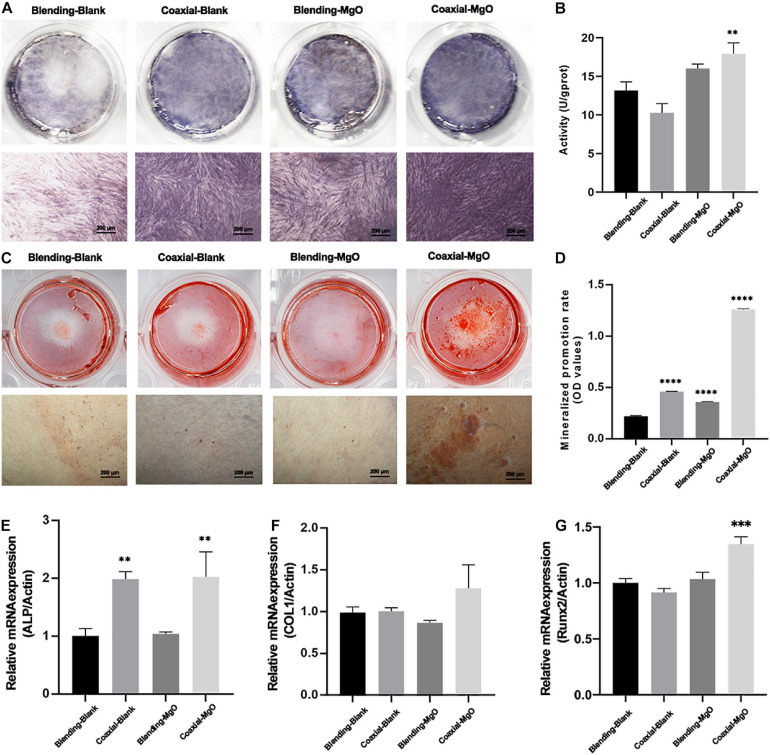
Effects of electrospun membranes on ALP activity and mineralization. **(A)** ALP staining of hPDLSCs after cultured with electrospun membranes for 7 days. **(B)** ALP activity level of hPDLSCs cocultured with electrospun membranes on the 7th day. **(C)** Alizarin red staining of hPDLSCs after seeded on electrospun membranes for 14 days. **(D)** Mineralized promotion rates of cells cultured with each group of the electrospun membranes. RT-qPCR analysis of **(E)** ALP, **(F)** COL1, and **(G)** Runx-2 expression of hPDLSCs after cultured with electrospun membranes for 3 days.

Osteogenic differentiation of hPDLSCs treated with PCL/gelatin nanocellulose membranes were further tested by detecting ALP activity, accumulated mineralized nodules, and expression of osteogenesis-related genes. Based on the ALP staining (Figure 5A), after 7 days of treatment, the expression of ALP showed obvious up-regulation in group Coaxial-MgO compared with other groups. ALP activity level on day 7 demonstrated similar trends, as shown in Figure 5B. These results indicated that Coaxial-MgO nanocellulose membranes could promise a satisfactory effect on the early osteogenic differentiation of hPDLSCs.

The formation of mineralized nodules was investigated by ARS staining. As shown in Figure 5C, after co-culturing with hPDLSCs for 14 days, Coaxial-MgO displayed the most obvious effect on mineralization compared with other groups. After being dissolved in 10% (w/v) cetylpyridinium chloride, the mineralized nodules were quantified by measuring the absorbance of ARS deposits at 562 nm, and the results showed the same trend (Figure 5D). The effects of PCL/gelatin nanocellulose membranes on osteogenic-related genes, including ALP, COL1, and Runx2, were further investigated by RT-qPCR, as shown in Figures 5E–G. After culturing for 3 days, Coaxial-MgO demonstrated the strongest ability in accelerating these mRNA expression levels. For Coaxial-Blank and Coaxial-MgO, the expression of ALP and Runx2 significantly rose, while there was no significant difference for COL1. Taken together, the results unarguably confirmed that Coaxial-MgO had stronger effects on osteogenic differentiation of hPDLSCs.

### Antimicrobial Activity of PCL/Gelatin Nanocellulose Membranes

**FIGURE 6:**
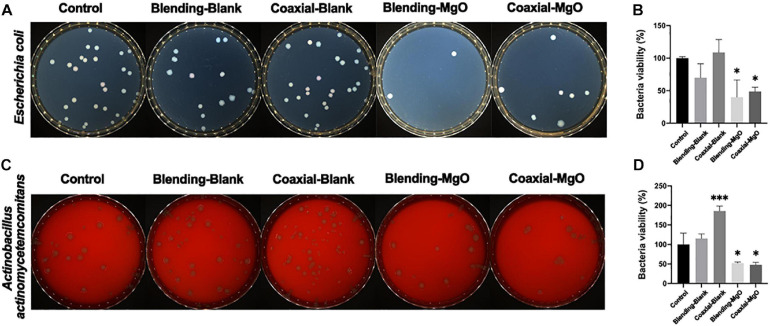
Antibacterial activity of various kinds of electrospun membranes against *E. coli* and *A. a.* Digital pictures of bacterial colony forming units and their corresponding quantitative bacterial survival rate for **(A,B)** *E. coli* and **(C,D)** *A. a.*

In order to understand the antibacterial activities of as-prepared PCL/gelatin nanocellulose membranes, *in vitro* antibacterial assay was carried out using *E. coli* and *A. a* as model. Colonies of *E. coli* (Figure 6A) and *A. a* (Figure 6C) significantly decreased when treated with Coaxial-MgO and Blending-MgO, which demonstrated the high antibacterial activities compared with Coaxial-Blank and Blending-Blank. This might be explained by the fact that the added MgO nanoparticles can improve these nanocellulose membranes’ antibacterial properties. Quantitative evaluation of bacterial colonies followed the same trends as shown in Figures 6B,D.

## Discussion

The loss of alveolar bone caused by periodontitis is a common clinical problem (Dentino et al., 2013). While the GTR technique widely uses membrane materials as a barrier in periodontal flap surgery, it still has some limitations, such as poor osteogenic and antimicrobial properties (Liang et al., 2020). To endow these biodegradable membrane with excellent osteogenic and antimicrobial activities, there are several emerging approaches (Naahidi et al., 2017; Prado-Prone et al., 2020; Shkodenko et al., 2020), among which MgO nanoparticles-incorporated PCL/gelatin nanocellulose membranes fabricated by coaxial electrospinning might be very promising platforms for periodontal tissue regeneration. Our results indicated that Coaxial-MgO had good biocompatibility and affinity to cells due to its gelatin shell, and incorporation of MgO nanoparticles could enhance the mechanical property of membrane. The release of Mg^2+^ is relatively sustainable for Coaxial-MgO, resulting in superior osteogenic and antimicrobial properties compared with Blending-Blank and Coaxial-Blank.

To evaluate the morphology of PCL/gelatin electrospinning nanocellulose membranes, TEM and SEM were analyzed. The results showed that Coaxial-Blank and Coaxial-MgO showed obvious core-shell fiber structure compared with Blending-Blank and Blending-MgO, which was in agreement with previous reports (Wang and Windbergs, 2019). From the TEM images, the MgO nanoparticles were successfully incorporated into the core layer, which was also confirmed by EDS. The distinct core-shell nanocellulose structure with the incorporation of MgO nanoparticles provides the foundation for further cell behavior and antibacterial activity assessment. Moreover, the thermal stability and tensile strength were also affected after the incorporation of MgO nanoparticles. These results were in accordance with Suryavanshi’ previous work (Suryavanshi et al., 2017). Nanocellulose membranes fabricated by the coaxial electrospinning method demonstrated relatively superior hydrophilicity compared with that of the single-nozzle electrospinning method, which might be attributed to the success of its gelatin shell. It is well known that Magnesium (Mg^2+^) has multiple essential roles in biological cells, and Mg^2+^ can regulate various cell behaviors by activating different signal pathways (Leem et al., 2016; Wang et al., 2017; Wang Z. et al., 2020). For example, Mg^2+^ may regulate the adhesion, proliferation, differentiation of human osteoblast via the TRPM7/PI3K pathway (Zhang et al., 2017). Besides, it has also been reported that Mg^2+^ can promote osteogenic differentiation of human bone marrow stromal cells (hBMSCs) by regulating Wnt, MAPK, and other signal pathways (Hung et al., 2019). Nevertheless, the regulation induced by Mg^2+^ was strictly dose-dependent (Yoshizawa et al., 2014), where the excessive Mg^2+^ could inhibit proliferation or osteogenic differentiation of cells. In our studies, compared with Blending-MgO, a more sustainable release of Mg^2+^ was observed in Coaxial-MgO, thus preventing their initial burst release. The core-shell fiber structure with high hydrophilicity and appropriate release of Mg^2+^ might have an increasingly important role in periodontal tissue engineering.

The GTR membranes are used to prevent epithelial migration into the regenerating area, allowing the slower migrating cells like periodontal ligament cells to occupy the defect site. The interactions between as-prepared nanocellulose membranes and hPDLSCs were investigated. Coaxial-MgO exhibited optimal adhesion and proliferation of hPDLSCs. More cells were attached on Coaxial-MgO’s surface, probably because the gelatin shell with great hydrophilicity improved cell adhesion (Liu et al., 2018). Similar positive effects of gelatin on the adhesion and proliferation of cells were also observed in another study (Yue et al., 2015). It has been reported that the excessive Mg^2+^ might be cytotoxic to biological cells (Mangalampalli et al., 2018; Onder et al., 2018). The initial burst release of Mg^2+^ from Blending-MgO might affect the proliferation of hPDLSCs. The effects of nanocellulose membranes on osteogenic differentiation of hPDLSCs were further assessed. There is accumulating evidence that magnesium-based materials were capable of accelerating the osteogenic activity of cells (Liu W. et al., 2019; Huang Y.-z et al., 2020; Li et al., 2020). The expression of ALP activity and formation of mineralized nodules of hPDLSCs treated with Coaxial-MgO were higher than those treated with the other membranes.

Additionally, Coaxial-MgO also significantly improved the expression of osteogenic-related genes, such as ALP and Runx2. Hence, Coaxial-MgO with high hydrophilicity and sustainable release of Mg^2+^ is beneficial for GTR membranes due to its facilitation in adhesion, proliferation, and osteogenic differentiation of hPDLSCs. The numbers of colonies could be directly visualized from the plate to assess the antibacterial activity. The decrease of colonies in the group Coaxial-MgO was statistically significant, which indicated that the fiber membrane carrying MgO nanoparticles had a certain antibacterial effect. MgO nanoparticles have been confirmed to be effective against pathogenic microorganisms (Nguyen et al., 2018). Generally, the neat nano-cellulose film is absent antimicrobial activity (Huang C. et al., 2020; Xiang et al., 2020; Nie et al., 2021). In this work, both of the prepared films have the antibacterial effect, in which Blending-MgO was more obvious. It is reasonable to speculate that the antibacterial effect might differ due to the preparation method of fiber membranes, which needs to be further studied.

## Summary

MgO nanoparticles-incorporated PCL/gelatin nanocellulose membranes were successfully fabricated using the coaxial electrospinning method. The obtained Coaxial-MgO membranes showed improved hydrophilicity and Mg^2+^ release kinetics compared with that of Blending-MgO. Coaxial-MgO also exhibited optimal proliferation, attachment, and osteogenic differentiation of hPDLSCs. In addition, the prepared Coaxial-MgO showed considerable antibacterial activity by prohibiting the growth of *E. coli* and *A. a.* The MgO nanoparticles-incorporated PCL/gelatin nanocellulose membranes fabricated by coaxial electrospinning with excellent osteogenic and antibacterial properties may be used as GTR membranes, which will bring innovation in the field of periodontal regeneration.

## Data Availability Statement

The original contributions presented in the study are included in the article/Supplementary Material, further inquiries can be directed to the corresponding author/s.

## Author Contributions

YX and SR conceptualized the study. WP performed the experiments. YZha helped to complete part of the experiment. Qing Jiang and WP examined the physicochemical properties of the membranes. RF contributed to the antibacterial evaluation of electrospun nanocellulose membranes. All authors contributed to the article and approved the submitted version.

## Conflict of Interest

The authors declare that the research was conducted in the absence of any commercial or financial relationships that could be construed as a potential conflict of interest.

## References

[B1] Dayem A. A. Choi H. Y. Yang G. M. Kim K. Saha S. K. Kim J. H. (2016). The potential of nanoparticles in stem cell differentiation and further therapeutic applications. *Biotechnol. J.* 11 1550–1560. 10.1002/biot.201600453 27797150

[B2] Dentino A. Lee S. Mailho J. Hefti A. F. (2013). Principles of periodontology. *Periodontology* 2000 61 16–53. 10.1111/j.1600-0757.2011.00397.x 23240942

[B3] De-Paula M. M. M. Afewerki S. Viana B. C. Webster T. J. Lobo A. O. Marciano F. R. (2019). Dual effective core-shell electrospun scaffolds: promoting osteoblast maturation and reducing bacteria activity. *Mater. Sci. Eng. C Mater. Biol. Appl.* 103:109778. 10.1016/j.msec.2019.109778 31349506

[B4] Dong H. Zheng L. Yu P. Jiang Q. Wu Y. Huang C. (2020). Characterization and application of lignin-carbohydrate complexes from lignocellulosic materials as antioxidants for scavenging in vitro and in vivo reactive oxygen species. *ACS Sustain. Chem. Eng.* 8 256–266. 10.1021/acssuschemeng.9b05290

[B5] Doostmohammadi M. Forootanfar H. Ramakrishna S. (2020). Regenerative medicine and drug delivery: progress via electrospun biomaterials. *Mater. Sci. Eng. C Mater. Biol. Appl.* 109:110521. 10.1016/j.msec.2019.110521 32228899

[B6] Graziani F. Karapetsa D. Alonso B. Herrera D. (2017). Nonsurgical and surgical treatment of periodontitis- how many options for one disease? *Periodontology* 2000 75 152–188. 10.1111/prd.12201 28758300

[B7] Gu J. Guo M. Huang C. Wang X. Zhu Y. Wang L. (2021). Titanium dioxide nanoparticle affects motor behavior, neurodevelopment and axonal growth in zebrafish (*Danio rerio*) larvae. *Sci. Total Environ.* 754:142315. 10.1016/j.scitotenv.2020.142315 33254858

[B8] Hickey D. J. Muthusamy D. Webster T. J. (2017). Electrophoretic deposition of MgO nanoparticles imparts antibacterial properties to poly-L-lactic acid for orthopedic applications. *J. Biomed. Mater. Res. A* 105 3136–3147. 10.1002/jbm.a.36174 28782240

[B9] Hiep N. Lee B. (2010). Electro-spinning of PLGA/PCL blends for tissue engineering and their biocompatibility. *J. Mater. Sci. Mater. Med.* 21 1969–1978. 10.1007/s10856-010-4048-y 20232234

[B10] Huang C. Dong H. Zhang Z. Bian H. Yong Q. (2020). Procuring the nano-scale lignin in prehydrolyzate as ingredient to prepare cellulose nanofibril composite film with multiple functions. *Cellulose* 27 9355–9370. 10.1007/s10570-020-03427-9

[B11] Huang C. Tang S. Zhang W. Tao Y. Lai C. Li X. (2018). Unveiling the structural properties of lignin-carbohydrate complexes in bamboo residues and its functionality as antioxidants and immunostimulants. *ACS Sustain. Chem. Eng.* 6 12522–12531. 10.1021/acssuschemeng.8b03262

[B12] Huang Y.-z Ji Y.-r Kang Z.-w Li F. Ge S.-f Yang D.-P. (2020). Integrating eggshell-derived CaCO3/MgO nanocomposites and chitosan into a biomimetic scaffold for bone regeneration. *Chem. Eng. J.* 395:125098. 10.1016/j.cej.2020.125098

[B13] Hung C. C. Chaya A. Liu K. Verdelis K. Sfeir C. (2019). The role of magnesium ions in bone regeneration involves the canonical Wnt signaling pathway. *Acta Biomater.* 98 246–255. 10.1016/j.actbio.2019.06.001 31181262

[B14] Karthik K. Dhanuskodi S. Gobinath C. Prabukumar S. Sivaramakrishnan S. (2019). Fabrication of MgO nanostructures and its efficient photocatalytic, antibacterial and anticancer performance. *J. Photochem. Photobiol. B* 190 8–20. 10.1016/j.jphotobiol.2018.11.001 30453162

[B15] Ke D. Tarafder S. Vahabzadeh S. Bose S. (2019). Effects of MgO, ZnO, SrO, and SiO2 in tricalcium phosphate scaffolds on in vitro gene expression and in vivo osteogenesis. *Mater. Sci. Eng. C Mater. Biol. Appl.* 96 10–19. 30606515 10.1016/j.msec.2018.10.073 PMC6484851

[B16] Kennedy K. M. Bhaw-Luximon A. Jhurry D. (2017). Cell-matrix mechanical interaction in electrospun polymeric scaffolds for tissue engineering: implications for scaffold design and performance. *Acta Biomater.* 50 41–55. 10.1016/j.actbio.2016.12.034 28011142

[B17] Khalf A. Madihally S. V. (2017). Recent advances in multiaxial electrospinning for drug delivery. *Eur. J. Pharm. Biopharm.* 112 1–17. 10.1016/j.ejpb.2016.11.010 27865991

[B18] Khandaker M. Li Y. Morris T. (2013). Micro and nano MgO particles for the improvement of fracture toughness of bone-cement interfaces. *J. Biomech.* 46 1035–1039. 10.1016/j.jbiomech.2012.12.006 23332232 PMC3594577

[B19] Larsson L. Decker A. M. Nibali L. Pilipchuk S. P. Berglundh T. Giannobile W. V. (2016). Regenerative medicine for periodontal and peri-implant diseases. *J. Dent. Res.* 95 255–266. 10.1177/0022034515618887 26608580 PMC4766955

[B20] Lee S. Matsugaki A. Kasuga T. Nakano T. (2019). Development of bifunctional oriented bioactive glass/poly(lactic acid) composite scaffolds to control osteoblast alignment and proliferation. *J. Biomed. Mater. Res. A* 107 1031–1041. 10.1002/jbm.a.36619 30675975 PMC6593822

[B21] Leem Y. H. Lee K. S. Kim J. H. Seok H. K. Chang J. S. Lee D. H. (2016). Magnesium ions facilitate integrin alpha 2- and alpha 3-mediated proliferation and enhance alkaline phosphatase expression and activity in hBMSCs. *J. Tissue Eng. Regen. Med.* 10 E527–E536. 10.1002/term.1861 24616281

[B22] Li C. Sun J. Shi K. Long J. Li L. Lai Y. (2020). Preparation and evaluation of osteogenic nano-MgO/PMMA bone cement for bone healing in a rat critical size calvarial defect. *J. Mater. Chem. B* 8 4575–4586. 10.1039/d0tb00074d 32242606

[B23] Li L. He Z. Y. Wei X. W. Wei Y. Q. (2016). Recent advances of biomaterials in biotherapy. *Regen. Biomater.* 3 99–105. 10.1093/rb/rbw007 27047675 PMC4817323

[B24] Li X. Kanjwal M. A. Lin L. Chronakis I. S. (2013). Electrospun polyvinyl-alcohol nanofibers as oral fast-dissolving delivery system of caffeine and riboflavin. *Colloids Surf. B Biointerfaces* 103 182–188. 10.1016/j.colsurfb.2012.10.016 23201736

[B25] Liang Y. Luan X. Liu X. (2020). Recent advances in periodontal regeneration: a biomaterial perspective. *Bioact. Mater.* 5 297–308. 10.1016/j.bioactmat.2020.02.012 32154444 PMC7052441

[B26] Lim S. H. Mao H. Q. (2009). Electrospun scaffolds for stem cell engineering. *Adv. Drug Deliv. Rev.* 61 1084–1096. 10.1016/j.addr.2009.07.011 19647024

[B27] Lin N. Menicanin D. Mrozik K. Gronthos S. Bartold P. (2008). Putative stem cells in regenerating human periodontium. *J. Periodontal Res.* 43 514–523. 10.1111/j.1600-0765.2007.01061.x 18624941

[B28] Lin Z. Wu J. Qiao W. Zhao Y. Wong K. H. M. Chu P. K. (2018). Precisely controlled delivery of magnesium ions thru sponge-like monodisperse PLGA/nano-MgO-alginate core-shell microsphere device to enable in-situ bone regeneration. *Biomaterials* 174 1–16. 10.1016/j.biomaterials.2018.05.011 29763774

[B29] Liu J. Ruan J. Weir M. D. Ren K. Schneider A. Wang P. (2019). Periodontal bone-ligament-cementum regeneration via scaffolds and stem cells. *Cells* 8:537. 10.3390/cells8060537 31167434 PMC6628570

[B30] Liu W. Zou Z. Zhou L. Liu H. Wen W. Zhou C. (2019). Synergistic effect of functionalized poly(l-lactide) with surface-modified MgO and chitin whiskers on osteogenesis in vivo and in vitro. *Mater. Sci. Eng. C Mater. Biol. Appl.* 103:109851. 10.1016/j.msec.2019.109851 31349474

[B31] Liu X. He X. Jin D. Wu S. Wang H. Yin M. (2020). A biodegradable multifunctional nanonanocellulose membrane for periodontal tissue regeneration. *Acta Biomater.* 108 207–222. 10.1016/j.actbio.2020.03.044 32251784

[B32] Liu Z. Tang M. Zhao J. Chai R. Kang J. (2018). Looking into the future: toward advanced 3D biomaterials for stem-cell-based regenerative medicine. *Adv. Mater.* 30:e1705388. 10.1002/adma.201705388 29450919

[B33] Lu Y. Tao P. Zhang N. Nie S. (2020). Preparation and thermal stability evaluation of cellulose nanofibrils from bagasse pulp with differing hemicelluloses contents. *Carbohydr. Polym.* 245:116463. 10.1016/j.carbpol.2020.116463 32718602

[B34] Mangalampalli B. Dumala N. Perumalla Venkata R. Grover P. (2018). Genotoxicity, biochemical, and biodistribution studies of magnesium oxide nano and microparticles in albino wistar rats after 28-day repeated oral exposure. *Environ. Toxicol.* 33 396–410. 10.1002/tox.22526 29282847

[B35] Meireles A. B. Correa D. K. da Silveira J. V. Millas A. L. Bittencourt E. de Brito-Melo G. E. (2018). Trends in polymeric electrospun fibers and their use as oral biomaterials. *Exp. Biol. Med. (Maywood)* 243 665–676. 10.1177/1535370218770404 29763386 PMC6378505

[B36] Monzavi A. Eshraghi S. Hashemian R. Momen-Heravi F. (2014). In vitro and ex vivo antimicrobial efficacy of nano-MgO in the elimination of endodontic pathogens. *Clin. Oral Investig.* 19 349–356. 10.1007/s00784-014-1253-y 24859291

[B37] Naahidi S. Jafari M. Logan M. Wang Y. Yuan Y. Bae H. (2017). Biocompatibility of hydrogel-based scaffolds for tissue engineering applications. *Biotechnol. Adv.* 35 530–544. 10.1016/j.biotechadv.2017.05.006 28558979

[B38] Needleman I. Worthington H. V. Giedrys-Leeper E. Tucker R. (2019). WITHDRAWN: guided tissue regeneration for periodontal infra-bony defects. *Cochrane Database Syst. Rev.* 5:CD001724. 10.1002/14651858.CD001724.pub3 31141165 PMC6541039

[B39] Ng J. Hynes K. White G. Sivanathan K. Vandyke K. Bartold P. (2016). Immunomodulatory properties of induced pluripotent stem cell-derived mesenchymal cells. *J. Cell. Biochem.* 117 2844–2853. 10.1002/jcb.25596 27167148

[B40] Nguyen N.-Y. T. Grelling N. Wetteland C. L. Rosario R. Liu H. (2018). Antimicrobial activities and mechanisms of magnesium oxide nanoparticles (nMgO) against pathogenic bacteria, yeasts, and biofilms. *Sci. Rep.* 8:16260. 10.1038/s41598-018-34567-5 30389984 PMC6214931

[B41] Nie S. Fu Q. Lin X. Zhang C. Lu Y. Wang S. (2021). Enhanced performance of a cellulose nanofibrils-based triboelectric nanogenerator by tuning the surface polarizability and hydrophobicity. *Chem. Eng. J.* 404:126512. 10.1016/j.cej.2020.126512

[B42] Nie S. Zhang C. Zhang Q. Zhang K. Zhang Y. Tao P. (2018a). Enzymatic and cold alkaline pretreatments of sugarcane bagasse pulp to produce cellulose nanofibrils using a mechanical method. *Ind. Crops Prod.* 124 435–441. 10.1016/j.indcrop.2018.08.033

[B43] Nie S. Zhang K. Lin X. Zhang C. Yan D. Liang H. (2018b). Enzymatic pretreatment for the improvement of dispersion and film properties of cellulose nanofibrils. *Carbohydr. Polym.* 181 1136–1142. 10.1016/j.carbpol.2017.11.020 29253942

[B44] Onder S. Calikoglu-Koyuncu A. C. Kazmanli K. Urgen M. Kok F. N. Torun-Kose G. (2018). Magnesium doping on TiN coatings affects mesenchymal stem cell differentiation and proliferation positively in a dose-dependent manner. *Biomed. Mater. Eng.* 29 427–438. 10.3233/BME-181000 30282341

[B45] Oortgiesen D. A. W. Plachokova A. S. Geenen C. Meijer G. J. Walboomers X. F. van den Beucken J. J. J. P. (2012). Alkaline phosphatase immobilization onto Bio-Gide^®^ and Bio-Oss^®^ for periodontal and bone regeneration. *J. Clin. Periodontol.* 39 546–555. 10.1111/j.1600-051X.2012.01877.x 22519301

[B46] Pant B. Park M. Park S. J. (2019). Drug delivery applications of core-sheath nanofibers prepared by coaxial electrospinning: a review. *Pharmaceutics* 11:305. 10.3390/pharmaceutics11070305 31266186 PMC6680404

[B47] Parham S. Kharazi A. Z. Bakhsheshi-Rad H. R. Ghayour H. Ismail A. F. Nur H. (2020). Electrospun nano-fibers for biomedical and tissue engineering applications: a comprehensive review. *Materials (Basel)* 13:2153. 10.3390/ma13092153 32384813 PMC7254207

[B48] Pei W. Chen Z. S. Chan H. Y. E. Zheng L. Liang C. Huang C. (2020). Isolation and identification of a novel anti-protein aggregation activity of lignin-carbohydrate complex from *Chionanthus retusus* leaves. *Front. Bioeng. Biotechnol.* 8:573991. 10.3389/fbioe.2020.573991 33102457 PMC7546364

[B49] Prado-Prone G. Silva-Bermudez P. Bazzar M. Focarete M. L. Rodil S. E. Vidal-Gutierrez X. (2020). Antibacterial composite membranes of polycaprolactone/gelatin loaded with zinc oxide nanoparticles for guided tissue regeneration. *Biomed. Mater.* 15:035006. 10.1088/1748-605X/ab70ef 31995538

[B50] Qian Y. Zhou X. Zhang F. Diekwisch T. G. H. Luan X. Yang J. (2019). Triple PLGA/PCL scaffold modification including silver impregnation, collagen coating, and electrospinning significantly improve biocompatibility, antimicrobial, and osteogenic properties for orofacial tissue regeneration. *ACS Appl. Mater. Interfaces* 11 37381–37396. 10.1021/acsami.9b07053 31517483 PMC7220812

[B51] Ren K. Wang Y. Sun T. Yue W. Zhang H. (2017). Electrospun PCL/gelatin composite nanofiber structures for effective guided bone regeneration membranes. *Mater. Sci. Eng. C Mater. Biol. Appl.* 78 324–332. 10.1016/j.msec.2017.04.084 28575991

[B52] Seo B.-M. Miura M. Gronthos S. Mark Bartold P. Batouli S. Brahim J. (2004). Investigation of multipotent postnatal stem cells from human periodontal ligament. *Lancet* 364 149–155. 10.1016/S0140-6736(04)16627-0 15246727

[B53] Shkodenko L. Kassirov I. Koshel E. (2020). Metal oxide nanoparticles against bacterial biofilms: perspectives and limitations. *Microorganisms* 8:1545. 10.3390/microorganisms8101545 33036373 PMC7601517

[B54] Si J. Cui Z. Wang Q. Liu Q. Liu C. (2016). Biomimetic composite scaffolds based on mineralization of hydroxyapatite on electrospun poly(ε-caprolactone)/nanocellulose fibers. *Carbohydr. Polym.* 143 270–278. 10.1016/j.carbpol.2016.02.015 27083369

[B55] Sridhar R. Lakshminarayanan R. Madhaiya K. Veluchamy A. B. Lim K. H. C. Ramakrishna S. (2014). Electrosprayed nanoparticles and electrospun nanofibers based on natural materials- applications in tissue regeneration, drug delivery and pharmaceuticals. *Chem. Soc. Rev.* 44:790–814. 10.1039/c4cs00226a 25408245

[B56] Stavropoulos A. Bertl K. Spineli L. M. Sculean A. Cortellini P. Tonetti M. (2020). Medium- and long-term clinical benefits of periodontal regenerative/reconstructive procedures in intrabony defects: systematic review and network meta-analysis of randomized controlled clinical studies. *J. Clin. Periodontol.* 48:410–430. 10.1111/jcpe.13409 33289191 PMC7986220

[B57] Suryavanshi A. Khanna K. Sindhu K. R. Bellare J. Srivastava R. (2017). Magnesium oxide nanoparticle-loaded polycaprolactone composite electrospun fiber scaffolds for bone–soft tissue engineering applications: in-vitro and in-vivo evaluation. *Biomed. Mater.* 12:055011. 10.1088/1748-605X/aa792b 28944766

[B58] Szentivanyi A. Chakradeo T. Zernetsch H. Glasmacher B. (2011). Electrospun cellular microenvironments: understanding controlled release and scaffold structure. *Adv. Drug Deliv. Rev.* 63 209–220. 10.1016/j.addr.2010.12.002 21145932

[B59] Wang J. Ma X.-Y. Feng Y.-F. Ma Z.-S. Ma T.-C. Zhang Y. (2017). Magnesium ions promote the biological behaviour of rat calvarial osteoblasts by activating the PI3K/Akt signalling pathway. *Biol. Trace Elem. Res.* 179 284–293. 10.1007/s12011-017-0948-8 28205079

[B60] Wang J. Windbergs M. (2019). Controlled dual drug release by coaxial electrospun fibers – impact of the core fluid on drug encapsulation and release. *Int. J. Pharm.* 556 363–371. 10.1016/j.ijpharm.2018.12.026 30572080

[B61] Wang P. Yin B. Dong H. Zhang Y. Zhang Y. Chen R. (2020). Coupling biocompatible au nanoclusters and cellulose nanofibrils to prepare the antibacterial nanocomposite films. *Front. Bioeng. Biotechnol.* 8:986. 10.3389/fbioe.2020.00986 32974314 PMC7466770

[B62] Wang Z. Liu Q. Liu C. Tan W. Tang M. Zhou X. (2020). Mg(2+) in beta-TCP/Mg-Zn composite enhances the differentiation of human bone marrow stromal cells into osteoblasts through MAPK-regulated Runx2/Osx. *J. Cell. Physiol.* 235 5182–5191. 10.1002/jcp.29395 31742679

[B63] Xiang Z. Jin X. Huang C. Li L. Wu W. Qi H. (2020). Water cast film formability of sugarcane bagasse xylans favored by side groups. *Cellulose* 27 7307–7320. 10.1007/s10570-020-03291-7

[B64] Xue J. He M. Liu H. Niu Y. Crawford A. Coates P. D. (2014). Drug loaded homogeneous electrospun PCL/gelatin hybrid nanofiber structures for anti-infective tissue regeneration membranes. *Biomaterials* 35 9395–9405. 10.1016/j.biomaterials.2014.07.060 25134855

[B65] Yoshizawa S. Brown A. Barchowsky A. Sfeir C. (2014). Magnesium ion stimulation of bone marrow stromal cells enhances osteogenic activity, simulating the effect of magnesium alloy degradation. *Acta Biomater.* 10 2834–2842. 10.1016/j.actbio.2014.02.002 24512978

[B66] Yue K. Trujillo-de Santiago G. Alvarez M. M. Tamayol A. Annabi N. Khademhosseini A. (2015). Synthesis, properties, and biomedical applications of gelatin methacryloyl (GelMA) hydrogels. *Biomaterials* 73 254–271. 10.1016/j.biomaterials.2015.08.045 26414409 PMC4610009

[B67] Zhang X. Zu H. Zhao D. Yang K. Tian S. Yu X. (2017). Ion channel functional protein kinase TRPM7 regulates Mg ions to promote the osteoinduction of human osteoblast via PI3K pathway: in vitro simulation of the bone-repairing effect of Mg-based alloy implant. *Acta Biomater.* 63 369–382. 10.1016/j.actbio.2017.08.051 28882757

[B68] Zhang Y. Wang P. Mao H. Zhang Y. Zheng L. Yu P. (2021). PEGylated gold nanoparticles promote osteogenic differentiation in in vitro and in vivo systems. *Mater. Des.* 197:109231. 10.1016/j.matdes.2020.109231

[B69] Zheng L. Yu P. Zhang Y. Wang P. Yan W. Guo B. (2021). Evaluating the bio-application of biomacromolecule of lignin-carbohydrate complexes (LCC) from wheat straw in bone metabolism via ROS scavenging. *Int. J. Biol. Macromol.* 176 13–25. 10.1016/j.ijbiomac.2021.01.103 33482216

[B70] Zwahlen R. A. Cheung L. K. Zheng L. W. Chow R. L. Li T. Schuknecht B. (2009). Comparison of two resorbable membrane systems in bone regeneration after removal of wisdom teeth: a randomized-controlled clinical pilot study. *Clin. Oral Implants Res.* 20 1084–1091. 10.1111/j.1600-0501.2009.01751.x 19751357

