# Supplementary material for: MgO Nanoparticles-Incorporated PCL/Gelatin-Derived Coaxial Electrospinning Nanocellulose Membranes for Periodontal Tissue Regeneration
Source: Front Bioeng Biotechnol. 2021 Mar 25;9:668428. doi: 10.3389/fbioe.2021.668428 (PMC8026878; doi:10.3389/fbioe.2021.668428)
Supplement: Supplementary file 1 [file Data_Sheet_1.docx]

**Supporting Information**

MgO Nanoparticles-incorporated PCL/gelatin-derived Coaxial Electrospinning nanocellulose membranes for Periodontal Tissue Regeneration

**Wenzao Peng^1,2†^, Shuangshuang Ren^1,2†^, Yibo Zhang^3^, Ruyi Fan^1,2^, Yi Zhou^1,2^, Lu Li^1,2^, Xuanwen Xu^1,2^, Yan Xu^1,2*^**

^1^Jiangsu Key Laboratory of Oral Diseases, Nanjing Medical University; Department of Periodontics, Affiliated Hospital of Stomatology, Nanjing Medical University, Nanjing, China

^2^Jiangsu Province Engineering Research Center of Stomatological Translational Medicine，Nanjing, China

^3^State Key Laboratory of Pharmaceutical Biotechnology, Department of Sports Medicine and Adult Reconstructive Surgery, Nanjing Drum Tower Hospital, The Affiliated Hospital of Nanjing University Medical School, Nanjing, China

**^†^** Wenzao Peng and Shuangshuang Ren contributed equally to this work, regarding as the first author.





**Figure S1.** TEM image of MgO nanoparticles.


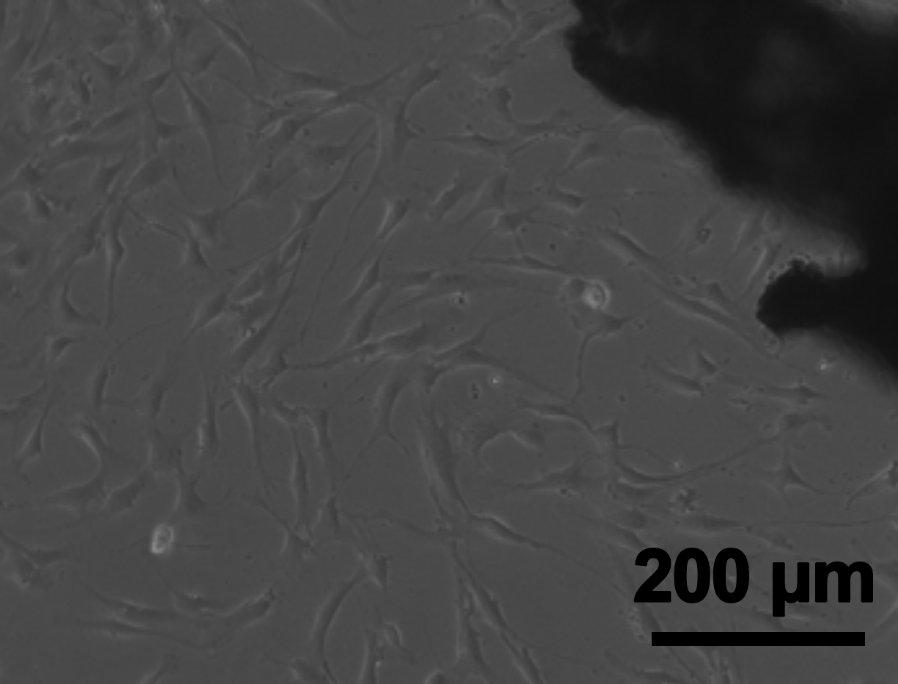


**Figure S2.** Photograph of the as-isolated hPDLSCs.


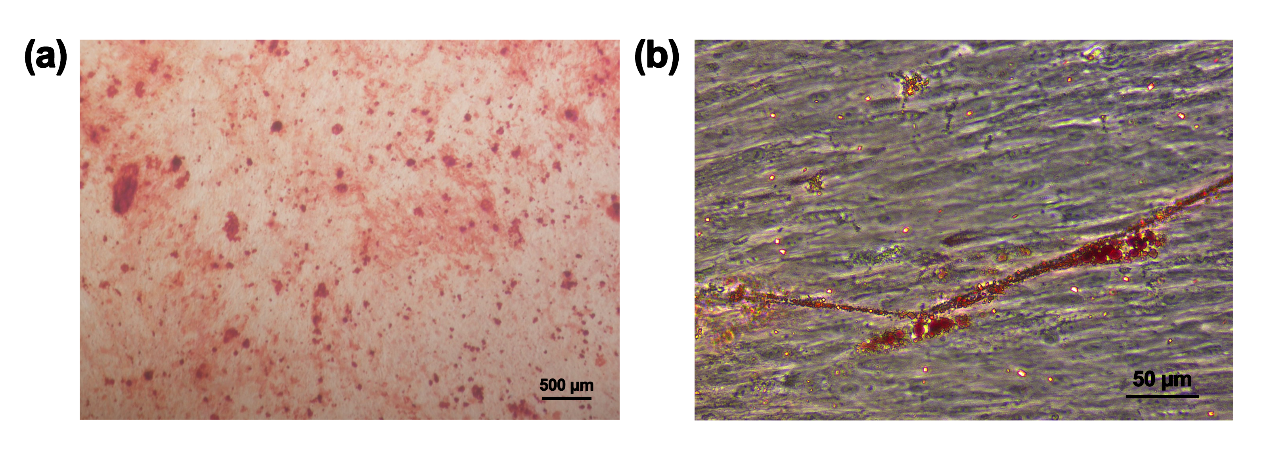


**Figure S3.** Osteogenic and adipogenic assay of hPDLSCs. (a)Mineralized nodules stained by ARS. (b) C Lipid clusters stained positive by Oil Red O.


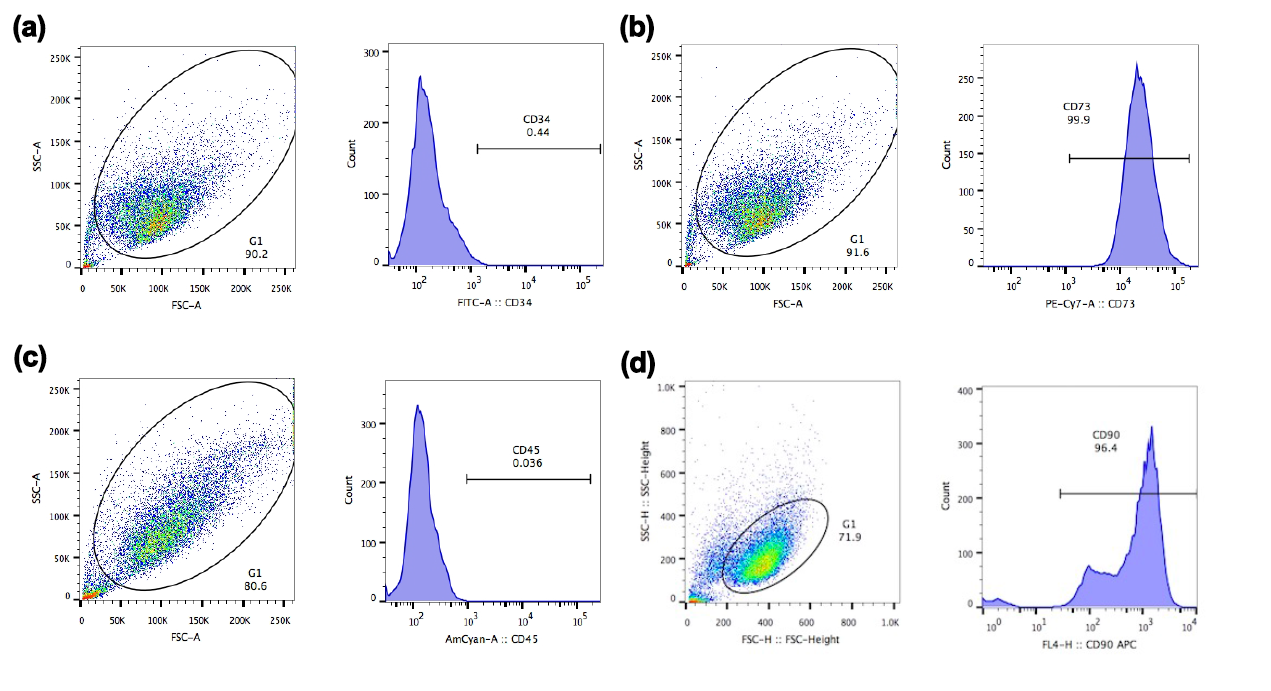


**Figure S4.** Flow cytometric analysis of hPDLSCs.


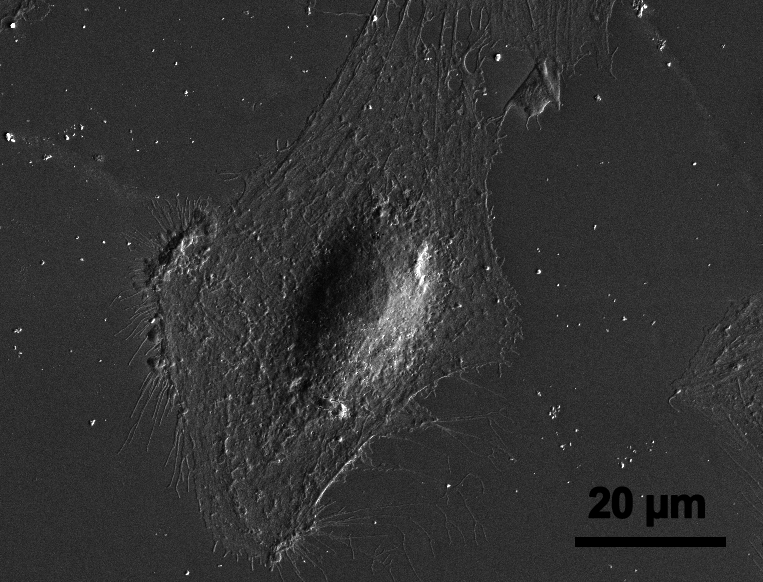


**Figure S5.** SEM image of hPDLSCs cultured on a round coverslip for 24h.


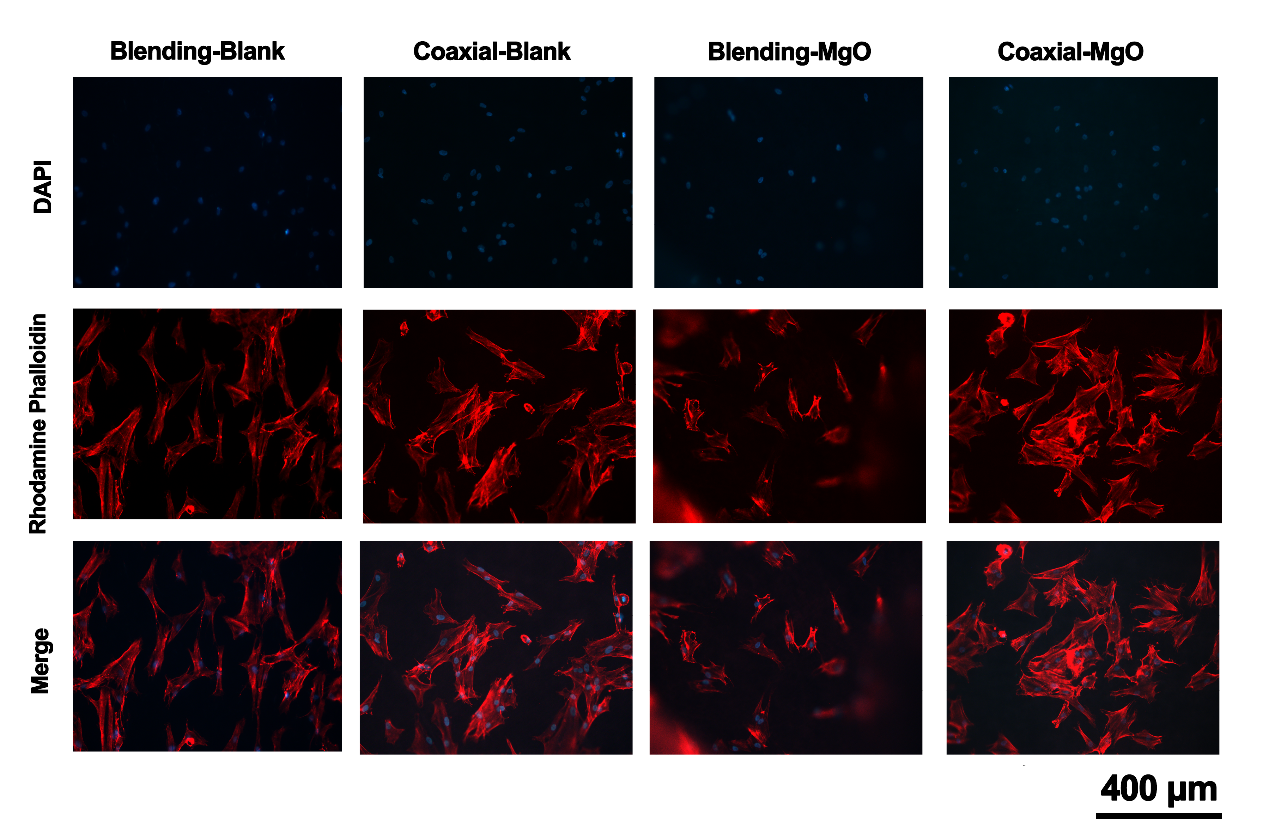


**Figure S6.** Fluorescent staining of hPDLSCs cultured on electrospun membranes for 12h, Blue: nuclei stained by DAPI; Red: cytoskeleton stained by rhodamine phalloidin.
